# A Multifunctional Dual-Luminescent Polyoxometalate@Metal-Organic Framework EuW_10_@UiO-67 Composite as Chemical Probe and Temperature Sensor

**DOI:** 10.3389/fchem.2018.00425

**Published:** 2018-09-24

**Authors:** William Salomon, Anne Dolbecq, Catherine Roch-Marchal, Grégoire Paille, Rémi Dessapt, Pierre Mialane, Hélène Serier-Brault

**Affiliations:** ^1^Institut Lavoisier de Versailles, UMR CNRS 8180, Université Paris-Saclay, Université de Versailles Saint-Quentin, Versailles, France; ^2^Laboratoire de Chimie des Processus Biologiques, UMR CNRS 8229, Collège de France, Université Pierre et Marie Curie, PSL Research University, Paris, France; ^3^Institut des Matériaux Jean Rouxel, CNRS, Université de Nantes, Nantes, France

**Keywords:** polyoxometalate, metal-organic framework, sensor, ratiometric luminescent thermometer, europium

## Abstract

The luminescent [EuW_10_O_36_]^9−^ polyoxometalate has been introduced into the cavities of the highly porous zirconium luminescent metal-organic framework UiO-67 via a direct synthesis approach, affording the EuW_10_@UiO-67 hybrid. Using a combination of techniques (TGA, BET, elemental analysis, EDX mapping,…) this new material has been fully characterized, evidencing that it contains only 0.25% in europium and that the polyoxometalate units are located inside the octahedral cavities and not at the surface of the UiO-67 crystallites. Despite the low amount of europium, it is shown that EuW_10_@UiO-67 acts as a solid-state luminescent sensor for the detection of amino-acids, the growth of the emission intensity globally following the growth of the amino-acid pKa. In addition, EuW_10_@UiO-67 acts as a sensor for the detection of metallic cations, with a high sensitivity for Fe^3+^. Noticeably, the recyclability of the reported material has been established. Finally, it is shown that the dual-luminescent EuW_10_@UiO-67 material behave as a self-calibrated-ratiometric thermometer in the physiological range.

## Introduction

Polyoxometalate (POM)-based photosystems are currently undergoing a remarkable development due to their high relevance in photocatalysis or their ability to act as efficient photoswitches or fluorescent probes (Han et al., 2015; Saad et al., 2015; Holmes-Smith et al., 2016; Chen et al., 2017; Natali et al., 2017). Focusing on this last topic, both materials based on purely inorganic POMs incorporating luminescent metallic centers and hybrid organic-inorganic entities bearing grafted organic optically active moieties have been elaborated. For example, fluorescent microspheres prepared from a Lindqvist POM covalently connected to pyrene groups have been used for the detection of foodborne pathogens (Ju et al., 2016). Inorganic tungsten and/or molybdenum POMs incorporating rare earth (RE) centers have also been shown to act as efficient luminescent probes. For instance, cryogenic optical thermal probes made of polyoxomolybdate building blocks incorporating Eu^III^ and Tb^III^ ions have been recently reported (Kaczmarek et al., 2017). The luminescence of such RE-incorporating POMs is also very sensitive to the chemical environment. This can be illustrated considering the works devoted to the interaction between proteins and the seminal [EuW_10_O_36_]^9−^ (EuW_10_) europium decatungstate characterized by narrow emission bands, a large Stokes shift and a long lifetime (Sugeta and Yamase, 1993). The characteristic emission of the europium decatungstate located at ca. 620 nm is strongly enhanced in presence of bovine or human serum albumin without any alteration of the secondary structure of the protein, showing that such simple molecular oxides could act as biological optical labeling agents (Hungerford et al., 2008; Zheng et al., 2010). Accordingly, Wu et al. showed that hybrid nanospheres made of arginine/lysine-rich peptides and EuW_10_ are characterized by a large Eu^III^ luminescence enhancement. Using a combination of techniques, it was evidenced that the observed phenomenon was due to the exclusion of the hydration water molecules from the secondary coordination sphere of EuW_10_ caused by the strong electrostatic and hydrogen bond interactions between POMs and basic amino-acids (AAs) (Zhang et al., 2015). Inversely, it was found that acidic AAs quench the luminescence while nonpolar AAs do not significantly influence the optical properties of the POM (Zhang et al., 2016). The processing of EuW_10_ by incorporation into films has been achieved, allowing the fabrication of moisture-responsive systems (Clemente-León et al., 2010; Xu et al., 2011; Qiu et al., 2015). Based on all these results, we have undertaken to elaborate a multi-functional hybrid system where the EuW_10_ POM is incorporated in the cavities of a metal-organic framework (MOF). This class of 3D coordination networks represents a unique platform for the development of solid-state luminescent materials due to their crystalline nature, permanent porosity, chemical tunability and robustness (Fernando-Soria et al., 2012; Hu et al., 2014; Lustig et al., 2017). To date, POM@MOF materials have been mainly synthesized for catalytic purposes. Two main synthetic strategies have been considered: (i) a one-pot strategy, where the precursors allowing the formation of the MOF and the POM are mixed all together, the MOF being built around the POM (direct synthesis) and (ii) the pre-formed MOF is impregnated with a solution of the POM (two-step strategy). Such materials have shown their efficiency for catalytic reactions ranging from the oxidation of alkylbenzene (Sun et al., 2016) to the C-H activation of nitrile (Shi et al., 2016) and water oxidation (Mukhopadhyay et al., 2018; Paille et al., 2018). Interestingly, it has also been shown that a single-molecule magnet iron POM isolated in the cavities of a diamagnetic MOF preserves its magnetic properties (Salomon et al., 2016). Besides, while Ln-MOFs and Ln-doped MOFs have been largely studied, the encapsulation of a well-defined inorganic luminescent compound in MOFs for sensing and detection has been rarely described (Cui et al., 2015; Wu et al., 2017). The white water-stable and highly porous zirconium MOF UiO-67 (Cavka et al., 2008) has been selected, and the ability of the EuW_10_@UiO-67 composite to act as sensor for metallic cations and AAs was investigated. Considering the dual-luminescent properties of EuW_10_@UiO-67—arising from both the inserted POMs and the 3D host network—its aptitude to act as a self-calibrated temperature sensor has also been explored.

## Experimental section

### Physical methods

Infrared (IR) spectra were recorded on a Nicolet 30 ATR 6700 FT spectrometer. Powder X-Ray diffraction data were obtained on a Bruker D5000 diffractometer using Cu radiation (1.54059 Å). C, H, N elemental analyses were performed by the Service de microanalyse of CNRS, 91198 Gif-sur-Yvette Cedex France. EDX measurements were performed on a JEOL JSM 5800 LV apparatus. Thermogravimetry analyses (TGA) were performed on a Mettler Toledo TGA/DSC 1, STARe System apparatus under oxygen flow (50 mL min^−1^) at a heating rate of 5°C min^−1^ up to 800°C. N_2_ adsorption isotherms were obtained at 77 K using a BELsorp Mini (Bel, Japan). Prior to the analysis, approximately 30 mg of sample were evacuated at 90°C under primary vacuum overnight. Room-temperature and temperature-dependant photoluminescence spectra were recorded on a Jobin-Yvon Fluorolog 3 fluorometer equipped with a CCD camera (excitation source: 450 W Xe arc lamp). The temperature was controlled by a nitrogen–closed cycle cryostat with vacuum system measuring and an Oxford Instrument ITC503S auto–tuning temperature controller with a resistance heater. The temperature can be adjusted from ca. 77 to 300 K with a maximum accuracy of 0.1 K. The sample temperature was fixed to a particular value using the auto–tuning temperature controller; after waiting 5 min to thermalize the sample, five consecutive steady–state emission spectra were measured for each temperature. The luminescence sensing experiments were carried out by introducing EuW_10_@UiO-67 powder (1.5 mg) into aqueous solutions (3 mL, 10^−2^ mol L^−1^) of MCl_x_ (M^n+^ = Na^+^, K^+^, Ni^2+^, Cr^3+^, Cu^2+^, Al^3+^, Mn^2+^, and Fe^3+^) or into MES/NaOH (MES = 2-(*N*-morpholino)ethanesulfonic acid) buffer solutions (pH = 6) of amino-acids (lysine, L-glycine, β-alanine, L-histidine, L-tryptophane, γ-aminobutyric acid, L-arginine) at room temperature. The mixtures were magnetically stirred during 5 min before collecting the luminescence data.

### Synthetic procedures

#### Chemicals and reagents

All available chemicals were purchased from major chemical suppliers and used as received. The Lindqvist-type europium decatungstate Na_9_[EuW_10_O_36_]·32H_2_O (EuW_10_) (Sugeta and Yamase, 1993) and the UiO-67 MOF (Salomon et al., 2015) have been synthesized as previously described.

#### Synthesis of DODA_9_[EuW_10_O_36_]·(DODACL)_2_ ((DODA)EuW_10_)

The dimethyldioctadecylammonium (DODA) salt of EuW_10_ was synthesized according to a procedure developed by L. Wu. et al. for (DODA)_13_H_2_[Eu(BW_11_O_39_)_2_]·25H_2_O (Li et al., 2008). A stoichiometric amount of DODA.Cl (1 equivalent by charge, 3.281 g, 5.6 mmol) was dissolved in 20 mL of chloroform. The solution was then added dropwise to a 10 mL aqueous solution of Na_9_[EuW_10_O_36_]·32H_2_O (2.082 g, 0.62 mmol) under stirring. The mixture was kept under vigorous stirring for 2 h. The organic phase was separated and dried with MgSO_4_. The solvent was then evaporated with a rotary evaporator to recover the DODA salt (3.9 g, yield = 72%). Anal. Calc. (found) for (DODA)_9_[EuW_10_O_36_]·(DODACl)_2_ (C_418_H_880_N_11_EuW_10_O_36_Cl_2_; M.W.: 8,699 g mol^−1^): C 57.71 (57.45), H 10.20 (10.51), N 1.77 (1.66). IR (ATR): ν (cm^−1^) 2,916 (s), 2,849 (s), 1,466 (m), 940 (m), 922 (m), 836 (m), 753 (s),718 (s). EDX measurements: atomic ratio calc. (exp.): W/Eu 10.0 (11.5).

#### Synthesis of [Zr_6_o_4_(OH)_4_][C_14_H_8_O_4_]_5.82_[EuW_10_O_36_]_0.04_·7H_2_O (EuW_10_@UiO-67)

ZrCl_4_ (116 mg, 0.5 mmol), biphenyl-dicarboxylic acid (121 mg, 0.5 mmol), (DODA)EuW_10_ (429 mg, 4.93 10^−5^ mol) and benzoic acid (1.83 g, 15 mmol) were briefly stirred in 10 mL of dimethylformamide (DMF) inside a 23 mL polytetrafluoroethylene-lined vessel. Hydrochloric acid 37% (83 μL) was added, and the mixture was heated at 120°C for 24 h. The solid was isolated by filtration of the hot mixture and thoroughly washed with DMF, chloroform, dry acetone and dried in an oven at 120°C overnight (yield: 118 mg, 60% based on Zr, 4% based on POM). Anal. Calc. (found) for [Zr_6_O_4_(OH)_4_][C_14_H_8_O_4_]_5.82_[EuW_10_O_36_]_0.04_·7H_2_O (C_81.2_H_64.4_O_39.64_Zr_6_W_0.4_Eu_0.04_; M.W.: 2,306 g mol^−1^): C 42.40 (42.50), H 2.82 (3.21). IR (ATR): ν (cm^−1^) 1,593 (m), 1,545 (m), 1,503 (w), 1,409 (s), 1,180 (w), 770 (m), 754 (w),736 (m), 704 (m), 669 (s), 455 (s). EDX measurements: atomic ratio calc. (exp.): Zr/W 15.0 (15.6), Eu/W 0.10 (0.13).

## Results and discussion

### Synthesis and characterization of the EuW_10_@UiO-67 material

**Figure 1:**
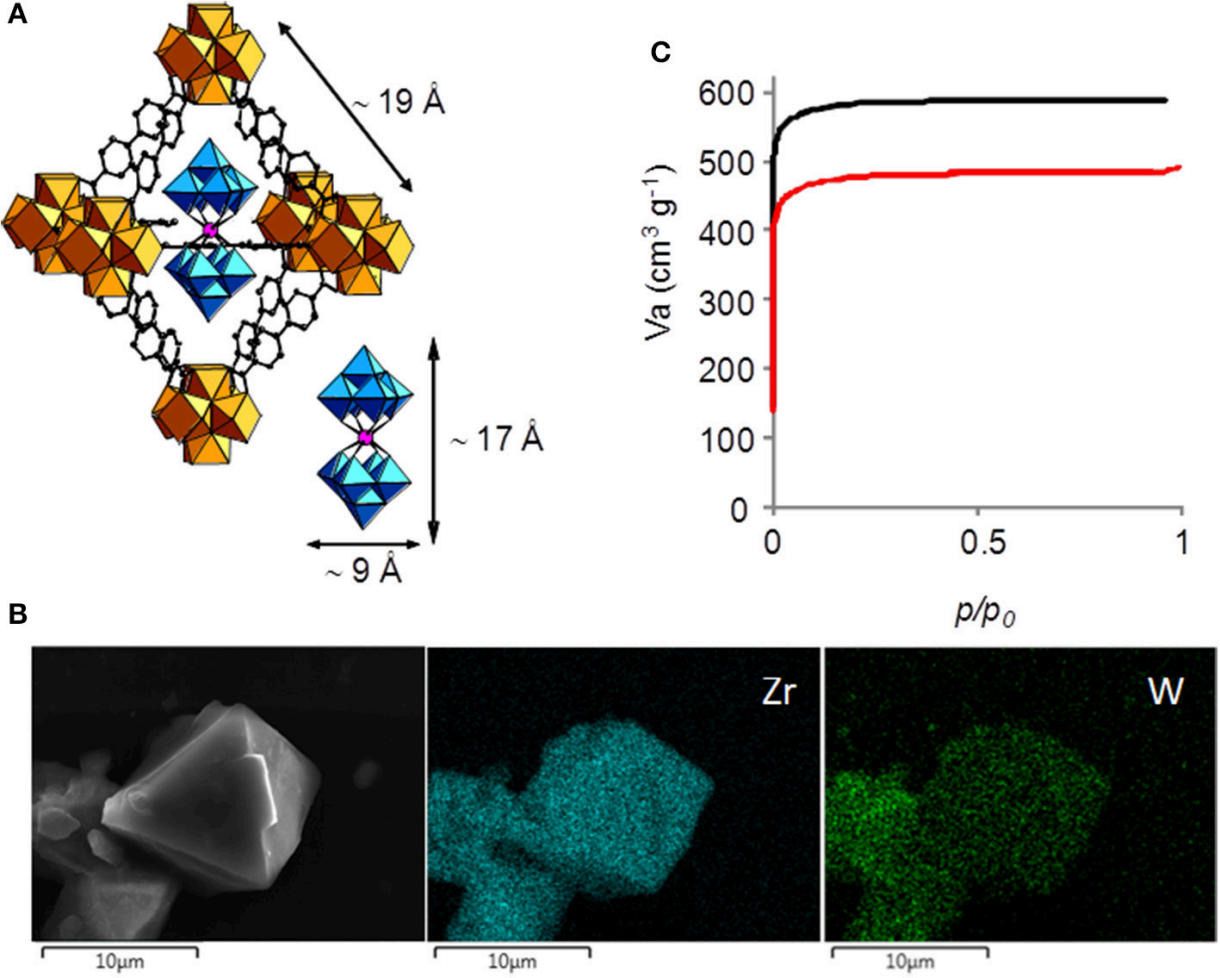
**(A)** Polyhedral representations of EuW_10_ and an octahedral cage of EuW_10_@UiO-67; blue octahedra: WO_6_, pink sphere: Eu, orange polyhedra: ZrO_8_, black sphere: C, **(B)** SEM image of crystals of EuW_10_@UiO-67 and EDS mapping for zirconium and tungsten, **(C)** N_2_ adsorption/desorption isotherms (77 K, *P*/*P*_0_ = 1 atm.) of UiO-67 (black) and the EuW_10_@UiO-67 composite (red).

We recently evidenced that the [PW_12_O_40_]^3−^ Keggin (Salomon et al., 2015), the [P_2_W_18_O_62_]^6−^ Dawson (Salomon et al., 2015) as well as the sandwich [(FeW_9_O_34_)_2_Fe_4_(H_2_O)_2_]^10−^ (Salomon et al., 2016) polyanions can be incorporated into the pores of the Zr(IV) biphenyldicarboxylate UiO-67 MOF. Considering the smallest dimensions of these POMs (ca. 12 Å) and the size of the triangular windows of the microporous MOF (ca. 8 Å), no impregnation of the molecular units into the preformed MOF could be envisaged and the POM@UiO-67 materials were obtained *via* a direct synthesis approach (Salomon et al., 2015, 2016). The same approach has been considered here, as the smallest dimension of EuW_10_ is ca. 9 Å. The title compound (Figure 1A) was thus synthesized by heating at 120°C in DMF a mixture of the dimethyldioctadecyl ammonium (DODA) salt of the POM and the precursors of UiO-67, affording EuW_10_@UiO-67 in good yield (60% based on Zr) as a white crystalline powder.

As observed in Figure S1, the experimental powder X-ray diffraction (PXRD) pattern of EuW_10_@UiO-67 and simulated PXRD pattern of UiO-67 are analogous, showing that the formation of the UiO-67 network can be obtained in the presence of the POM in the synthetic medium. In addition, the experimental PXRD pattern of the as-synthesized EuW_10_@UiO-67 and of EuW_10_@UiO-67 stirred for 1 h in water at room temperature are similar, showing that the hybrid is stable in such conditions. EDS mapping evidences both the presence of MOF and POM (Figure 1B). EDX measurements combined with elemental analysis allowed to propose for EuW_10_@UiO-67 the formula [Zr_6_O_4_(OH)_4_][C_14_H_8_O_4_]_5.82_[EuW_10_O_36_]_0.04_·7H_2_O, indicating that the material only contains ca. 0.25 wt % in europium. The TGA curve (Figure S2) reveals steps that are attributed to water removal (weight loss 5.0%, calculated 5.5%), linker decomposition and formation of inorganic oxides (weight loss 58.6%, calculated 60.5%). The IR spectrum (Figure S3) confirms that no DODA counterion is present in the MOF, the negative charges introduced by the POMs being compensated by linker deficiencies (Katz et al., 2013). Also, no vibration at ca. 1,680 cm^−1^ is observed, indicating that no free benzoic acid is present in the cavities of the MOF. In addition, it can be deduced from the formula that only 1/25 of the octahedral cavities are occupied by EuW_10_, this loading being lower than for [(FeW_9_O_34_)_2_Fe_4_(H_2_O)_2_]^10−^ (1/10) (Salomon et al., 2016). The N_2_ sorption isotherms experiments were conducted for both UiO-67 and EuW_10_@UiO-67 (Figure 1C and Table S1). As expected, the surface area is lower for EuW_10_@UiO-67 (S_BET_ = 1,900 m2.g^−1^) than for UiO-67 (S_BET_ = 2,400 m2.g^−1^), since a heavy compound representing 5% of the sample weight has been added. The value of the normalized specific surface area, taking into account the contribution of the mass of UiO-67 in EuW_10_@UiO-67 (2,400 × 0.95 = 2,280 m2.g^−1^), is significantly different from the experimental value of EuW_10_@UiO-67 (1,900 m2.g^−1^), suggesting that EuW_10_ is located inside the octahedral cavities and not at the surface of the UiO-67 particles (Table S1). In addition, it is observed that the porous distribution (Figure S4) is not significantly modified, evidencing only one peak since the hexagonal and tetrahedral cavities are connected by the same triangular windows (Barrett et al., 2012).

### Photophysical characterization of EuW_10_@UiO-67

**Figure 2:**
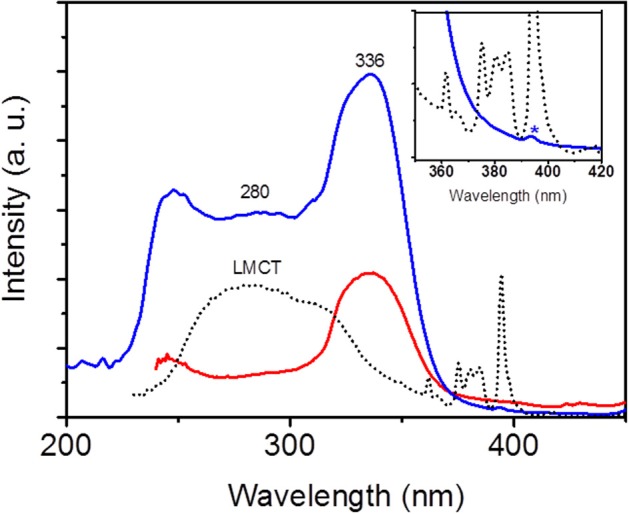
Room-temperature excitation spectra of EuW_10_@UiO-67 monitored at λ_em_ = 471 nm (red line) and at λ_em_ = 700 nm (blue line) and excitation spectrum of EuW_10_ (black dotted line) monitored at λ_em_ = 700 nm. Inset: the asterisk represents the ^7^F_0_→^5^L_6_ excitation band of the Eu^3+^ ion.

**Figure 3:**
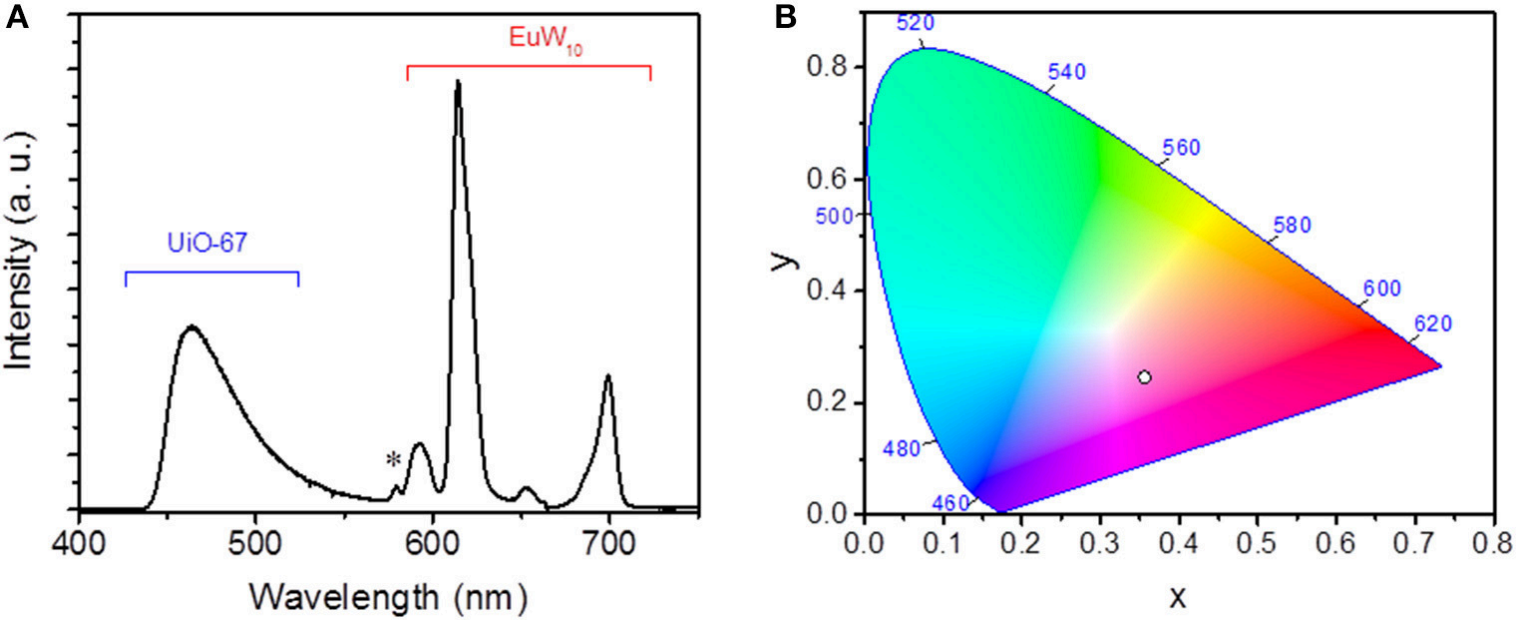
**(A)** Room-temperature emission spectrum monitored at λ_exc_ = 336 nm of EuW_10_@UiO-67. The asterisk highlights the ^5^D${}_{0}\to^{7}$F_0_ transition related to the Eu^3+^ ion; **(B)** CIE chromaticity diagram for EuW_10_@UiO-67 excited at 336 nm.

The photophysical properties of EuW_10_@UiO-67 were thoroughly investigated at room temperature. Firstly, the solid-state photoluminescence (PL) properties of the pure EuW_10_ and UiO-67 compounds were studied separately before considering the EuW_10_@UiO-67 composite. As expected, the excitation spectrum of EuW_10_ (Figure S5) is composed of a broad band centered at 280 nm, corresponding to the O → W ligand-to-metal charge transfer (LMCT) band, and the sharp lines characteristic of the f-f transitions of the Eu^3+^ ion (Yamase and Sugeta, 1993). Under photoexcitation at λ_exc_ = 280 nm, EuW_10_ shows a red luminescence (Figures S5 and S6) associated to the typical ^5^D_0_→^7^F_0−4_ transitions of Eu^3+^ ions in the 587–700 nm range, indicating an efficient intramolecular energy transfer from ligand to Eu^3+^. As this latter ion is located in a slightly distorted square antiprismatic site close to *D*_4d_ point symmetry, the ^5^D${}_{0}\to^{7}$F_2_ electric dipole transition (611–620 nm) gets a very weak intensity compared to that of the ^5^D${}_{0}\to^{7}$F_1_ magnetic dipole transition (Capobianco et al., 1990; Nogami and Abe, 1996). Upon UV excitation at 254 nm, the UiO-67 MOF exhibits a blue emission (Figure S6). Its PL spectrum under excitation at 336 nm (Figure S7) displays a broad band in the 450–600 nm range, with a maximum wavelength located at 471 nm, attributed to intraligand π → π^*^ transitions centered on the biphenyldicarboxylate linker. For the EuW_10_@UiO-67 composite, the excitation spectrum, monitored within the ^5^D_0_→^7^F_4_ (700 nm) Eu^3+^ transition (Figure 2), shows the LMCT broad band (λ_max_ = 280 nm) and the weakly intense sharp lines of the Eu^3+^ f-f transitions of EuW_10_ as well as the excitation bands of UiO-67 (λ_max_ = 336 nm). The presence of the LMCT band is a strong indication of the integrity of EuW_10_ in the composite, whereas the presence of the excitation bands of UiO-67, within the ^5^D_0_→^7^F_4_ Eu^3+^ transition, quite evidences an intermolecular MOF-to-Eu^3+^ energy transfer. Then, upon UV excitation at 336 nm, the emission spectrum of EuW_10_@UiO-67 corresponds to the association of the red emission of EuW_10_ together with the UiO-67 blue one (Figure 3), and hence, the composite is a good violet emitter with color coordinates *(x,y)* equal to x = 0.356 and y = 0.246. Moreover, the intensity ratio of the ^5^D${}_{0}\to^{7}$F_2_ transition to the ^5^D${}_{0}\to^{7}$F_1_ transition is significantly increased compared to that observed for pure EuW_10_. In addition, the symmetry-forbidden electric dipole ^5^D${}_{0}\to^{7}$F_0_ transition (580 nm) which does not appear in pure EuW_10_ is distinguishable in the PL spectrum of EuW_10_@UiO-67. These results unambiguously reveal that the site symmetry of the Eu^3+^ ion in EuW_10_ decreases when the POM is introduced into the MOF pores (Sugeta and Yamase, 1993).

### EuW_10_@UiO-67 as sensor for metallic cations and amino-acids

**Figure 4:**
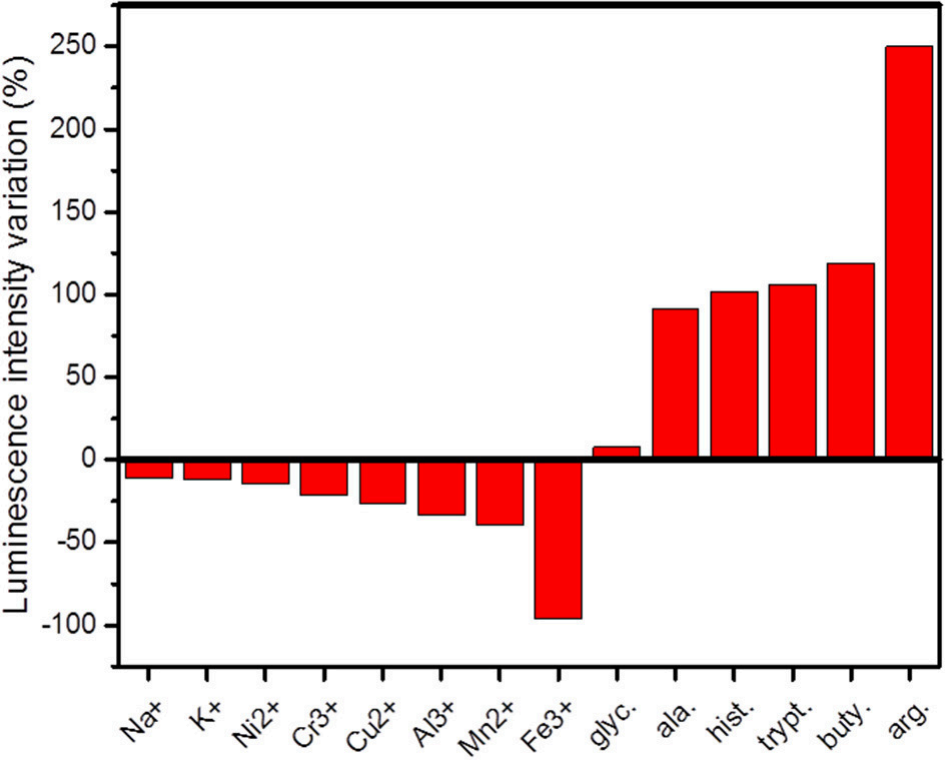
Luminescence intensity variations of the ^4^D${}_{0}\to^{7}$F_2_ transition (λ_exc_ = 336 nm) of EuW_10_@UiO-67 interacting with amino-acids MES/NaOH buffer solutions (glyc., glycine; ala., β-alanine; hist, L-histidine; trypt., L-tryptophan; buty., γ-aminobutyric acid; arg., L-arginine) and different metal ions aqueous solutions (C = 10^−2^ mol.L^−1^).

The luminescent sensing properties of EuW_10_@UiO-67 were first investigated for the detection of metal ions (Na^+^, K^+^, Ni^2+^, Cr^3+^, Cu^2+^, Al^3+^, Mn^2+^, and Fe^3+^) present in aqueous solutions (10^−2^ M) as chloride salts. The PL properties of suspensions of EuW_10_@UiO-67 into the metallic solutions (8.5 μM in Eu^3+^) were monitored at λ_exc_ = 336 nm and the luminescence intensity variations of the most intense ^5^D${}_{0}\to^{7}$F_2_ Eu^3+^ transition at 611 nm are depicted in Figure 4 and Figure S8a. The presence of all studied metal ions in solution systematically induces a decrease of the luminescence intensity of the Eu^3+^ f-f bands compared to their intensity when EuW_10_@UiO-67 is simply immersed in deionized water. The same effect is also observable on the organic ligand emission at 471 nm. Strikingly, Fe^3+^ ions can quench the whole emission of the composite, which becomes non-emissive under UV-light. This high sensitivity regarding Fe^3+^ ions offers an interesting perspective to use EuW_10_@UiO-67 as an efficient luminescent chemical probe for Fe^3+^ ions which play a relevant role in many biological processes (Xu and Yan, 2015). A study of the dependence of the quenching of the luminescence with the Fe^3+^ concentration was then conducted. The quenching effect can be quantitatively rationalized by the Stern-Volmer equation: I_0_/I = 1 + K_SV_[Fe^3+^], where I_0_ and I are the fluorescence intensities of the ^5^D_0_→^7^F_2_ transition of the Eu^3+^ ion in the EuW_10_@UiO-67 suspension in the absence or presence of Fe^3+^, respectively. K_SV_ is the quenching constant, which is calculated as 2,667 L.mol^−1^ with a very good linear correlation (R) of 0.999, indicating the strong quenching effect from Fe^3+^ (Figure S9). The concentration limit for the detection is estimated to be 37 μM, corresponding to the concentration for which the fluorescence intensity variation is <10%. To assess the robustness of the EuW_10_@UiO-67 composite, we have checked its recyclability after immersion into a Fe^3+^ aqueous solution (C = 10^−2^ M). The emission spectrum of the powder was monitored in the same conditions (Figure S10) before, directly after immersion, and after being washed several times with ultrapure water, filtered and dried under air. This study highlights that after washing, the emission of the composite practically recovered its initial intensity, quite evidencing that the EuW_10_@UiO-67 exhibits a robustness enabling to be reusable to detect Fe^3+^ ions in an aqueous solution. Nevertheless, while the XRD data of EuW_10_@UiO-67 after immersion in Fe^3+^ aqueous solution are in accordance with the simulated UiO-67 pattern, a certain loss of crystallinity is observed (Figure S11). In short, although the amount of the EuW_10_ emitter is weak into the MOF, the properties of the composite as a chemical probe is very promising with a recyclability, never investigated in these systems so far.

EuW_10_@UiO-67 was also tested as chemical probe for basic amino-acids (AAs), such as glycine, β-alanine, L-histidine, L-tryptophan, L-arginine, and γ-aminobutyric acid (Table S2). EuW_10_@UiO-67 (8.5 μM in Eu^3+^) was dispersed in MES/NaOH (pH = 6) buffer solutions of AAs (10^−2^ M) to further monitor the luminescence properties at λ_exc_ = 336 nm. The emission spectra are depicted in Figure S8b. As already noticed for the pure [Eu(SiW_10_MoO_39_)_2_]^13−^ POM in homogeneous aqueous solutions (Li et al., 2014), distinct luminescence enhancements are observed when the EuW_10_@UiO-67 material is in contact with the various AAs molecules (Figure 4). The arginine, which is the most basic AA of the investigated series, induces more than 2.5-fold enhancement of emission intensity. Moreover, the increase of the emission intensity globally follows the increase of the AA pKa and isoelectric point (Table S2). This is in line with the fact that the luminescence of Eu-based POMs is quenched by the surrounding water molecules, which are displaced in presence of protonated AAs due to electrostatic interactions and C-H···O contacts with the POM surface (Li et al., 2014). The recyclability of the EuW_10_@UiO-67 composite after exposure to the arginine solution has been also investigated. The emission spectrum of the powder was monitored in the same conditions (Figure S12) before, directly after immersion, and after being washed several times with ultrapure water, filtered and dried under air. This study highlights that after washing, the emission of the composite practically recovered its initial intensity. However, the XRD data of EuW_10_@UiO-67 after immersion in arginine aqueous solution also reveal a certain loss of crystallinity.

### EuW_10_@UiO-67 as self-calibrated-ratiometric luminescent thermometer

**Figure 5:**
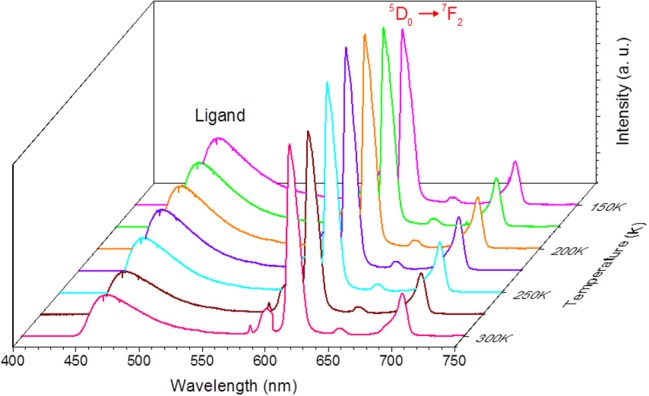
Emission spectra of compound EuW_10_@UiO-67 in the range 150–300 K with the excitation fixed at 336 nm.

As both the biphenyldicarboxylate linker of UiO-67 and EuW_10_ act as emitters, we further evaluated the capability of EuW_10_@UiO-67 to be used as a self-calibrated ratiometric luminescent thermometer. For that, the integrated areas of the ligand (*I*_*Ligand*_) and ^5^D_0_→^7^F_2_ Eu^3+^ (*I*_*Eu*_) emissions were used to define the thermometric parameter Δ = *I*_*Ligand*_/*I*_*Eu*_ permitting the conversion of emission intensities into absolute temperature. The temperature dependence of the EuW_10_@UiO-67 emission is presented in Figure 5 for the 150–300 K range. Five consecutive emission spectra were collected for each temperature and used to determine subsequent average data. The *I*_*Ligand*_ and *I*_*Eu*_ parameters have been obtained by integrating the emission spectra in the 438–575 nm and 604–630 nm wavelength intervals, respectively. The temperature dependence of the defined thermometric parameters Δ in the 200–300 K range is presented in Figure S13, where the solid line represents the temperature calibration curve. The parameter Δ decreases with the temperature according to the following empirical linear relationship:

Δ=1.985−0.0029T

with a correlation coefficient *R*2 of 0.998. The corresponding relative sensitivity, defined as *s*_*r*_ = |∂Δ/∂*T*|/Δ, and used as a figure of merit to compare the performance of distinct systems (Brites et al., 2016) is plotted in Figure S14, showing that EuW_10_@UiO-67 exhibits a maximum relative sensitivity S_m_ of ca. 0.26% K^−1^ for the physiological temperature range.

## Conclusion

In conclusion, we have reported herein an easy to prepare luminescent multifunctional material made of a metal-organic framework incorporating a europium-containing polyoxotungstate. Despite the very low amount of POM inserted in the UiO-67 matrix, it has been shown that EuW_10_@UiO-67 is able to act as a sensor ([Eu^3+^] = 8.5 μM) for metallic ions and amino-acids in water and in heterogeneous conditions. Among a series of cationic species, a good sensitivity has been found for Fe^3+^, while an enhancement of the EuW_10_@UiO-67 luminescence is observed in presence of amino-acids, the enhancement of the emission intensity globally following the increase of the amino-acid pKa. Moreover, the recyclability of EuW_10_@UiO-67 has been demonstrated, even if a certain loss of crystallinity is observed. In addition, the dual-luminescent properties–arising both from the POM and from the MOF - lead the reported material to behave as a self-calibrated ratiometric luminescent thermometer. Considering the huge number of luminescent POMs and MOFs already available, this first report of a dual-luminescent POM@MOF material thus opens the way to the development of such optical sensors.

## Author contributions

WS, AD, CR-M, PM and GP have performed the synthesis and the structural characterizations of the title compound. RD and HS-B have performed the photophysical characterizations. The manuscript was written by PM and HS-B with valuable contributions and corrections of CR-M and RD.

### Conflict of interest statement

The authors declare that the research was conducted in the absence of any commercial or financial relationships that could be construed as a potential conflict of interest.

## References

[B1] Barrett S. M. Wang C. Lin W. (2012). Oxygen sensing via phosphorescence quenching of doped metal–organic frameworks. J. Mater. Chem. 22, 10329–10334. 10.1039/c2jm15549d

[B2] Brites C. D. S. Millán A. Carlos L. D. (2016). Handbook on the Physics and Chemistry of Rare Earths. eds Bünzli J. C. Pecharsky V. K. Amsterdam: Elsevier.

[B3] Capobianco J. A. Proulx P. P. Bettinelli M. Negrisolo F. (1990). Absorption and emission spectroscopy of Eu^3+^ in metaphosphate glasses. Phys. Rev. B 42, 5936–5944. 9994667 10.1103/physrevb.42.5936

[B4] Cavka J. H. Jakobsen S. Olsbye U. Guillou N. Lamberti C. Bordiga S.. (2008). A new zirconium inorganic building brick forming metal organic frameworks with exceptional stability. J. Am. Chem. Soc. 130, 13850–13851. 10.1021/ja8057953 18817383

[B5] Chen S. Ma P. Luo H. Wang Y. Niu J. Wang J. (2017). A luminescent polyoxoniobate lanthanide derivative {Eu_3_(H_2_O)_9_[Nb_48_O_138_(H_2_O)_6_]}^27−^. Chem. Commun. 53, 3709–3712. 10.1039/C7CC00591A 28300244

[B6] Clemente-León M. Coronado E. López-Muñoz A. Repetto D. Ito T. Konya T.. (2010). Dual-emissive photoluminescent Langmuir–Blodgett films of decatungstoeuropate and an amphiphilic iridium complex. Langmuir 26, 1316–1324. 10.1021/la902513z 19754063

[B7] Cui Y. Song R. Yu J. Liu M. Wang Z. Wu C.. (2015). Dual-emitting MOF?dye composite for ratiometric temperature sensing. Adv. Mat. 27, 1420–1425. 10.1002/adma.201404700 25581401

[B8] Fernando-Soria J. Khajavi H. Serra-Crespo P. Gascon J. Kapteijin F. Julve M.. (2012). Highly selective chemical sensing in a luminescent nanoporous magnet. Adv. Mater. 24, 5625–5629. 10.1002/adma.201201846 22887721

[B9] Han X. B. Li Y. G. Zhang Z. M. Tan H. Q. Lu Y. Wang E. B. (2015). Polyoxometalate-based nickel clusters as visible light-driven water oxidation catalysts. J. Am. Chem. Soc. 137, 5486–5493. 10.1021/jacs.5b01329 25866996

[B10] Holmes-Smith A. S. Crisp J. Hussain F. Patzke G. R. Hungerford G. (2016). Use of lanthanide-containing polyoxometalates to sensitise the emission of fluorescent labeled serum albumin. ChemPhysChem 17, 418–424. 10.1002/cphc.201500954 26642428

[B11] Hu Z. Deibert B. J. Li J. (2014). Luminescent metal-organic frameworks for chemical sensing and explosive detection. Chem. Soc. Rev. 43, 5815–5840. 10.1039/C4CS00010B 24577142

[B12] Hungerford G. Suhling K. Green M. (2008). Luminescence enhancement of a europium containing polyoxometalate on interaction with bovine serum albumin. Photochem. Photobiol. Sci. 7, 734–737. 10.1039/b802793e 18528560

[B13] Ju W. Song X. Yan G. Xu K. Wang J. Yin D. (2016). Layer-by-layer assembly of polyoxometalate–pyrene-decorated fluorescent microspheres for the suspension immunoassay of Listeria monocytogenes. J. Mater. Chem. B, 4, 4287–4294. 10.1039/C6TB00986G 32263410

[B14] Kaczmarek A. M. Liu J. Laforce B. Vincze L. Van Hecke K. Van Deun R. (2017). Cryogenic luminescent thermometers based on multinuclear Eu^3+^/Tb^3+^ mixed lanthanide polyoxometalates. Dalton Trans. 46, 5781–5785. 10.1039/C7DT01058C 28401979

[B15] Katz M. J. Brown Z. J. Colón Y. J. Siu P. W. Scheidt K. A. Snurr R. Q.. (2013). A facile synthesis of UiO-66, UiO-67 and their derivatives. Chem. Commun. 49, 9449–9451. 10.1039/c3cc46105j 24008272

[B16] Li H. W. Wang Y. Zhang T. Wu Y. Wu L. (2014). Selective binding of amino acids on europium-substituted polyoxometalates and the interaction-induced luminescent enhancement effect. ChemPlusChem 79, 1208–12013. 10.1002/cplu.201402091

[B17] Li W. Yin S. Wang J. Wu L. (2008). Tuning mesophase of ammonium amphiphile-encapsulated polyoxometalate complexes through changing component structure. Chem. Mater. 20, 514–522. 10.1021/cm702955j

[B18] Lustig W. P. Mukherjee S. Rudd N. D. Desai A. V. Li J. Ghosh S. K. (2017). Metal-organic frameworks: functional luminescent and photonic materials for sensing applications. Chem. Soc. Rev. 46, 3242–3285. 10.1039/C6CS00930A 28462954

[B19] Mukhopadhyay S. Debgupta J. Singh C. Kar A. Das S. K. (2018). A Keggin polyoxometalate shows water oxidation activity at neutral pH: POM@ZIF-8, an efficient and Robust electrocatalyst. Angew. Chem. Int. Ed. 57, 1918–1923. 10.1002/anie.201711920 29240276

[B20] Natali M. Bazzan I. Goberna-Ferrón S. Al-Oweini R. Ibrahim M. Bassil B. S. (2017). Photo-assisted water oxidation by high-nuclearity cobalt-oxo cores: tracing the catalyst fate during oxygen evolution turnover. Green Chem. 19, 2416–2426. 10.1039/C7GC00052A

[B21] Nogami M. Abe Y. (1996). Properties of sol—gel-derived Al_2_O_3_-SiO_2_ glasses using Eu3+ion fluorescence spectra. J. Non-Cryst. Solid 197, 73–78.

[B22] Paille G. Gomez-Mingot M. Roch-Marchal C. Lassalle-Kaiser B. Mialane P. Fontecave M.. (2018). A fully noble metal-free photosystem based on cobalt-polyoxometalates immobilized in a porphyrinic metal-organic-framework for water oxidation. J. Am. Chem. Soc. 140, 3613–3618. 10.1021/jacs.7b11788 29393639

[B23] Qiu Y. F. Liu H. Zhang C. Ma Z. Hu P. A. Gao G. G. (2015). Moisture-responsive films consisting of luminescent polyoxometalates and agarose. J. Mater. Chem. C 3, 6322–6328. 10.1039/C5TC00421G

[B24] Saad A. Oms O. Dolbecq A. Menet C. Dessapt R. Serier-Brault H.. (2015). A high fatigue resistant, photoswitchable fluorescent spiropyran–polyoxometalate–BODIPY single-molecule. Chem. Commun. 51, 16088–16091. 10.1039/C5CC06217A 26390409

[B25] Salomon W. Lan Y. Rivière E. Yang S. Roch-Marchal C. Dolbecq A.. (2016). Single-molecule magnet behavior of individual polyoxometalate molecules incorporated within biopolymer or metal–organic framework matrices. Chem. Eur. J. 22, 6564–6574. 10.1002/chem.201600202 27080557

[B26] Salomon W. Roch-Marchal C. Mialane P. Rouschmeyer P. Serre C. Haouas M.. (2015). Immobilization of polyoxometalates in the Zr-based metal organic framework UiO-67. Chem. Commun. 51, 2972–2975. 10.1039/C4CC09986A 25594369

[B27] Shi D. He C. Sun W. Ming Z. Meng C. Duan C. (2016). A photosensitizing decatungstate-based MOF as heterogeneous photocatalyst for the selective C–H alkylation of aliphatic nitriles. Chem. Commun. 52, 4714–4717. 10.1039/C6CC00862C 26954389

[B28] Sugeta M. Yamase T. (1993). Crystal structure and luminescence site of Na_9_[EuW_10_O_36_]·32H_2_O. Bull. Chem. Soc. Jpn. 66, 444–449. 10.1246/bcsj.66.444

[B29] Sun J. W. Yan P. F. An G. H. Sha J. Q. Li G. M. Yang G. Y. (2016). Immobilization of polyoxometalate in the metal-organic framework rht-MOF-1: towards a highly effective heterogeneous catalyst and dye scavenger. Sci. Rep. 6:25595. 10.1038/srep25595 27157290 PMC4860640

[B30] Wu M. M. Wang J. Y. Sun R. Zhao C. Zhao J. P. Che G. B.. (2017). The design of dual-emissive composite material [Zn_2_(HL)_3_]^+^@MOF-5 as self-calibrating luminescent sensors of Al^3+^ ions and monoethanolamine. Inorg. Chem. 56, 9555–9562. 10.1021/acs.inorgchem.7b00939 28758753

[B31] Xu J. Zhao S. Han Z. Wang X. Song Y. F. (2011). Layer-by-layer assembly of Na_9_[EuW_10_O_36_]·32 H_2_O and layered double hydroxides leading to ordered ultra-thin films: cooperative effect and orientation effect. Chem. Eur. J. 17, 10365–10371. 10.1002/chem.201101062 21837721

[B32] Xu X. Y. Yan B. (2015). Eu(III)-functionalized MIL-124 as fluorescent probe for highly selectively sensing ions and organic small molecules especially for Fe(III) and Fe(II). ACS Appl. Mater. Interfaces 7, 721–729. 10.1021/am5070409 25510710

[B33] Yamase T. Sugeta M. (1993). Charge-transfer photoluminescence of polyoxo-tungstates and–molybdates. J. Chem. Soc. Dalton Trans. 1993, 759–765. 10.1039/dt9930000759

[B34] Zhang H. Guo L. Xie Z. Xin X. Sun D. Yuan S. (2016). Tunable aggregation-induced emission of polyoxometalates via amino acid-directed self-assembly and their application in detecting dopamine. Langmuir 32, 13736–13745. 10.1021/acs.langmuir.6b03709 27973851

[B35] Zhang T. Li H. W. Wu Y. Wang Y. Wu L. (2015). Self-assembly of an europium-containing polyoxometalate and the arginine/lysine-rich peptides from human papillomavirus capsid protein L1 in forming luminescence-enhanced hybrid nanoparticles. J. Phys. Chem. C 119, 8321–8328. 10.1021/acs.jpcc.5b00032

[B36] Zheng L. Ma Y. Zhang G. Yao J. Keita B. Nadjo L. (2010). A multitechnique study of europium decatungstate and human serum albumin molecular interaction. Phys. Chem. Chem. Phys. 12, 1299–1304. 10.1039/B919952G 20119607

