# Supplementary material for: A Multifunctional Dual-Luminescent Polyoxometalate@Metal-Organic Framework EuW10@UiO-67 Composite as Chemical Probe and Temperature Sensor
Source: Front Chem. 2018 Sep 24;6:425. doi: 10.3389/fchem.2018.00425 (PMC6165868; doi:10.3389/fchem.2018.00425)
Supplement: Supplementary file 1 [file Table_1.docx]

Electronic Supplementary Information

A multifunctional dual-luminescent polyoxometalate@metal-organic framework EuW_10_@UiO-67 composite as chemical probe and temperature sensor

William Salomon,^1^ Anne Dolbecq,^1^ Catherine Roch-Marchal,^1^ Grégoire Paille,^1,2^ Rémi Dessapt,^3^ Pierre Mialane*^1^ and Hélène Serier-Brault*^3^

^1^ Institut Lavoisier de Versailles, UMR CNRS 8180, Université Paris-Saclay, Université de Versailles Saint-Quentin, 45 avenue des Etats-Unis, 78035 Versailles Cedex, France

^2^ Laboratoire de Chimie des Processus Biologiques, UMR CNRS 8229, Collège de France, Université Pierre et Marie Curie, PSL Research University, 11 Place Marcelin Berthelot, 75231 Paris Cedex 05, France

^3^ Institut des Matériaux Jean Rouxel, Université de Nantes, CNRS, 2 rue de la Houssinière, BP 32229, 44322 Nantes, France

*** Correspondence:**Pierre Mialane [pierre.mialane@uvsq.fr](mailto:pierre.mialane@uvsq.fr), Hélène Serier-Brault helene.brault@cnrs-imn.fr

**I Additional physical characterizations of EuW_10_@UiO-67**

**Figure S1.** Experimental powder X-Ray diffraction patterns of as synthesized EuW_10_@UiO-67 (red) and EuW_10_@UiO-67 stirred for one hour in water at room temperature, filtered and dried (green); simulated PXRD pattern of UiO-67 (blue).

**Figure S2.** TGA curves of UiO-67 (black) and EuW_10_@UiO-67 (red).

**Figure S3.** IR spectrum of EuW_10_@UiO-67.

|  | S_BET_  (m^2^.g^-1^) | Total pore volume (cm^3^.g^-1^) |
| --- | --- | --- |
| UiO-67 | 2400 | 0.91 |
| EuW_10_@UiO-67 | 1900 | 0.75 |

**Table S1.** Specific surface area and total pore volumes for UiO-67 and EuW_10_@UiO-67.

**Figure S4.** Porous distribution for the UiO-67 MOF (black line) and the EuW_10_@UiO-67

composite (red).

**II Optical measurements**

**Figure S5.** Room-temperature excitation spectrum (black line) monitored at λ_em_ = 700 nm and emission spectrum (red line) monitored at λ_exc_ = 280 nm in the ligand-to-metal charge transfer (LMCT) band for EuW_10_.

**Figure S6.** Pictures of UiO-67 (left) and EuW_10_@UiO-67 (right) under visible light (top) and irradiated at 365 (middle) and 254 (bottom) nm.

**Figure S7.** Room-temperature excitation spectrum (black line) monitored at λ_em_ = 471 nm and emission spectrum (red line) monitored at λ_exc_ = 336 nm for the UiO-67 MOF.

**Fig. S8**. a) PL spectra monitored at λ_exc_ = 336 nm of EuW_10_@UiO-67 into different aqueous solutions ([Eu^3+^] = 8.5 μM) dispersed of various metal ions (10^-2^ M); b) PL spectra monitored at λ_exc_ = 336 nm of EuW_10_@UiO-67 dispersed into different aqueous solutions ([Eu^3+^] = 8.5 μM) of various amino acids (10^-2^ M).

**Fig. S9**. a) Emission spectra monitored at λ_exc_ = 336 nm of EuW_10_@UiO-67 dispersed into various concentrations of Fe^3+^ aqueous solutions; b) K_SV_ curve of EuW_10_@UiO-67 spectra dispersed into various concentrations of Fe^3+^ aqueous solutions, the I_0_/I ratio being calculated for the ^5^D_0_→^7^F_2_ Eu^3+^ transition (λ_exc_ = 336 nm).

**Figure S10.** Solid-state emission spectra monitored at λ_exc_ = 336 nm of EuW_10_@UiO-67 a) before dispersion into a Fe^3+^ solution (black line), b) dispersed into a Fe^3+^ ([Fe^3+^] = 10^-2^ M) aqueous solution (blue line) and c) after dispersion into a Fe^3+^ ([Fe^3+^] = 10^-2^ M) aqueous solution and thoroughly washed with water (red line).

**Figure S11.** Experimental powder X-Ray diffraction patterns of EuW_10_@UiO-67 after immersion into a Fe^3+^ (10^-2^ M) aqueous solution (red) and into a MES/NaOH buffer solution of arginine (10^-2^ M) (black); simulated PXRD pattern of UiO-67 (blue).

**Figure S12.** Solid-state emission spectra monitored at λ_exc_ = 336 nm of EuW_10_@UiO-67 a) before dispersion into an arginine solution (blue line), b) dispersed into water (black line), c) dispersed into a MES/NaOH (pH = 6) buffer solution of arginine (10^-2^ M) (red line) and d) after dispersion into a MES/NaOH (pH = 6) buffer solution of arginine (10^-2^ M) and thoroughly washed with water (green line).

**Figure S13.** Temperature dependence of Δ in the 200 − 300 K range for EuW_10_@UiO-67 excited at 336 nm. The line represents the calibration curve obtained by the best fit of the experimental points to the equation I_Ligand_/I_Eu_ = 1.985 – 0.0029 T (R^2^> 0.998).

**Figure S14.** Temperature dependence of the corresponding relative thermal sensitivities in the the 200 − 300 K range for EuW_10_@UiO-67 excited at 336 nm.

| **Nom** | **structure** | **pKa** | **Isoelectric point** |
| --- | --- | --- | --- |
| L-histidine | 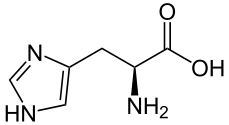 | 9.17 | 7.59 |
| L-tryptophane | 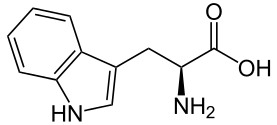 | 9.39 | 5.88 |
| β-alanine | 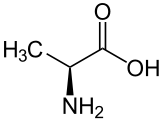 | 9.69 | 6.01 |
| glycine | 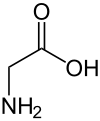 | 9.7 | 5.97 |
| γ-aminobutyric acid | 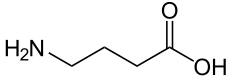 | 10.43 | 7.30 |
| L-arginine | 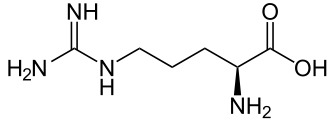 | 12.48 | 10.76 |

**Table S2**: List of investigated basic AAs with their structure, their pKa and their isoelectric point.
